# The Herald Bleed: A Case Report of an Aortoenteric Fistula causing an Acute Lower Gastrointestinal Bleed

**DOI:** 10.7759/cureus.6966

**Published:** 2020-02-12

**Authors:** Sundus Bhatti, Omer Endashaw, Jacolby Short

**Affiliations:** 1 Internal Medicine, University of Louisville School of Medicine, Louisville, USA; 2 Gastroenterology, University of Louisville, Louisville, USA; 3 Radiology, University of Louisville, Louisville, USA

**Keywords:** aortoenteric fistula, aortocolonic fistula, secondary aortoeneteric fistula, gi bleed, hematochezia

## Abstract

Aortoenteric fistula (AEF) is a rare life-threatening condition. Secondary AEF (SAEF) occurs in patients with abdominal aortic aneurysm (AAA) repair and has a high mortality rate. We present a case of a 66-year-old female who presented to the emergency room with hematochezia and hemodynamic instability. Emergent esophagogastroduodenoscopy (EGD) was negative. Colonoscopy revealed a 5 cm, pulsatile, bleeding, submucosal mass. A stat computed tomography (CT) angiogram of the abdomen and pelvis revealed a large left external iliac artery (LEIA) pseudoaneurysm. Vascular surgery emergently placed a LEIA stent. It appeared the patient had an aorto-bi-iliac (ABI) common iliac artery (CIA) bypass, 15 years ago, for a large AAA that had resulted in a SAEF, with the rare finding of communication with the colon. She had a complicated course involving surgical and medical management but with successful outcomes.

## Introduction

Aortoenteric fistula (AEF) is a rare but life-threatening condition with an annual incidence of 0.007/million [1]. Primary aortoenteric fistula (PAEF) arises de novo, whereas, secondary aortoenteric fistula (SAEF) occurs following abdominal aortic aneurysm (AAA) repair or aortic reconstruction [2-4]. The incidence of SAEF following aortic surgical reconstructions ranges from 0.36% to 1.6% [5]. AEF is more common in males compared with females, paralleling the incidence of AAA and aortic surgery. The male-to-female ratio is 3:1 for PAEF and 8:1 for SAEF [6]. Majority of primary and secondary AEFs involve the third and fourth portions of the duodenum, where the duodenum is in closest proximity to the aorta [1]. Other sites such as the colon are rarely involved (5%) [7]. Early diagnosis of AEF is difficult and depends on a heightened clinical suspicion. Clinical outcomes are determined by the timeliness of surgical repair, revascularization approach, type of surgery (emergent vs nonemergent), and occurrence of complications [2]. It may present as an exsanguinating herald gastrointestinal (GI) bleed, associated with high mortality rates. SAEF has a 45.8% mortality rate in the first month [8].

## Case presentation

A 66-year-old African American female presented to the emergency room with sudden onset of continuous large volume bright red blood per rectum. Due to her lethargy, limited history was obtained; she denied any prior episodes of GI bleed, alcohol or nonsteroidal anti-inflammatory drug use and had never undergone a prior upper or lower endoscopy. Past medical history was pertinent for hypertension, coronary artery disease, and stroke. Prior surgical interventions included cholecystectomy and hysterectomy. Her vitals indicated hemodynamic instability with a blood pressure of 70/45 mmHg, heart rate of 112 beats/minute. On physical examination, she was lethargic but arousable to voice, had abdominal tenderness and a midline abdominal incision which she stated was from her hysterectomy; there were no stigmata of chronic liver disease and digital rectal exam showed bright red blood in the rectum. Her laboratory workup was as follows: hemoglobin 9.5, hematocrit 29%, platelets 147,000, International Normalized Ratio (INR) 1, and low salicylate levels. Abdominal X-ray was unremarkable. 

**Figure 1:**
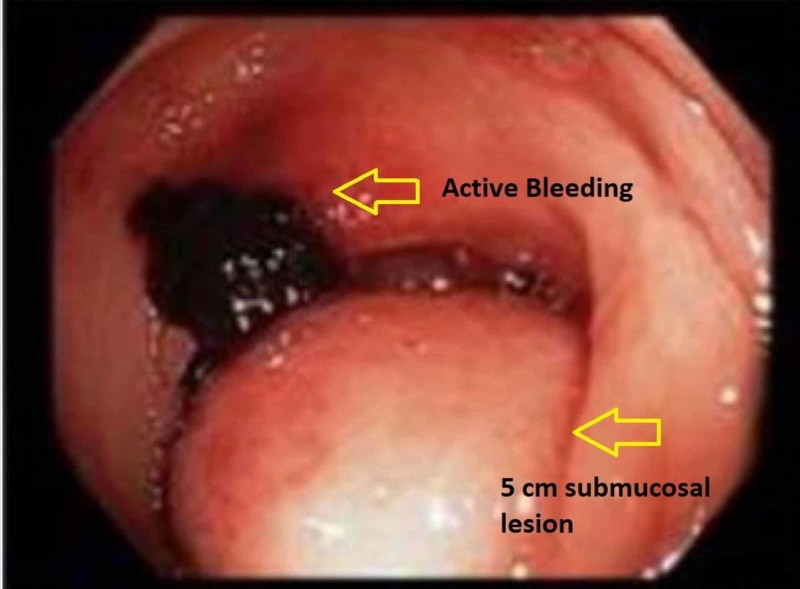
A pulsatile 5 cm submucosal mass in the sigmoid colon with an actively bleeding ulcerated area viewed during colonoscopy

**Figure 2:**
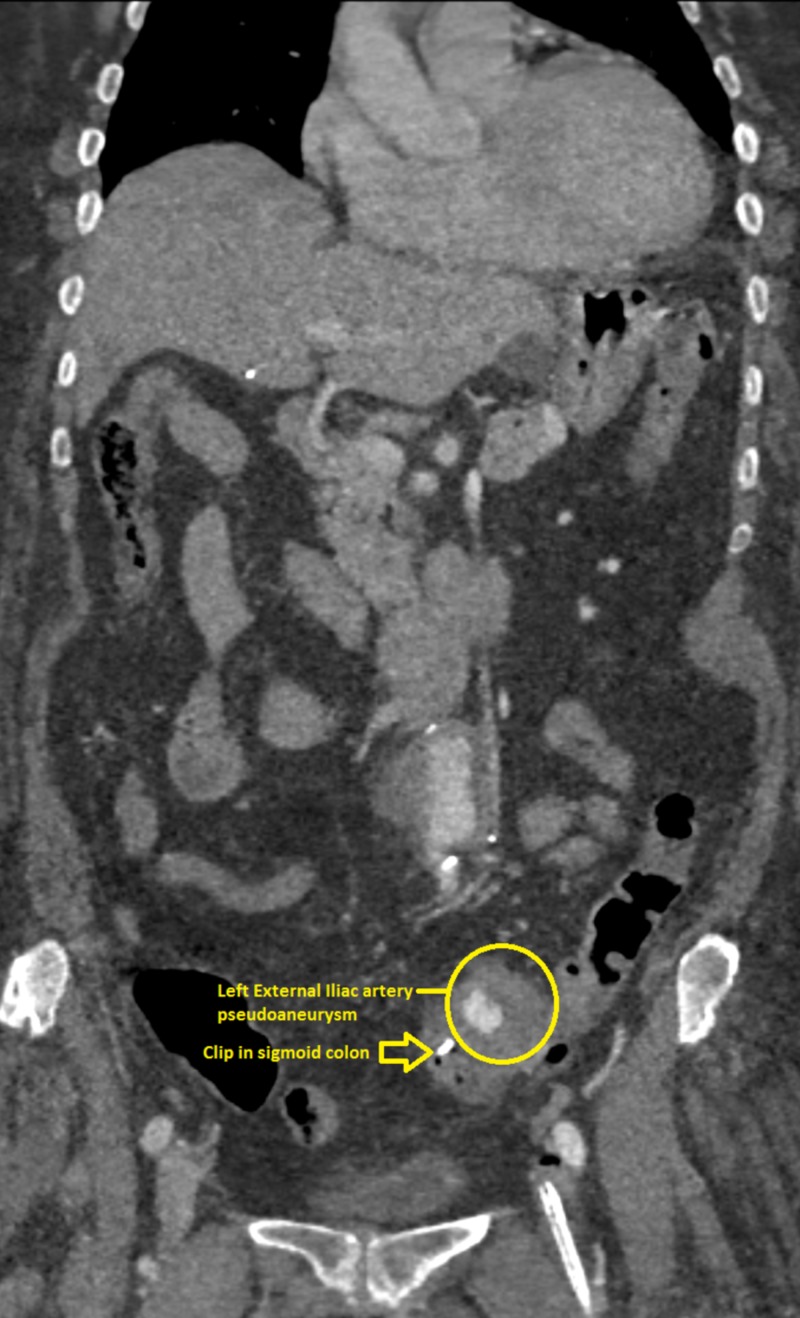
Coronal contrast-enhanced computed tomography (CT) image demonstrates endoscopically placed clip in sigmoid colon adjacent to the partially thrombosed left external iliac artery pseudoaneurysm

**Figure 3:**
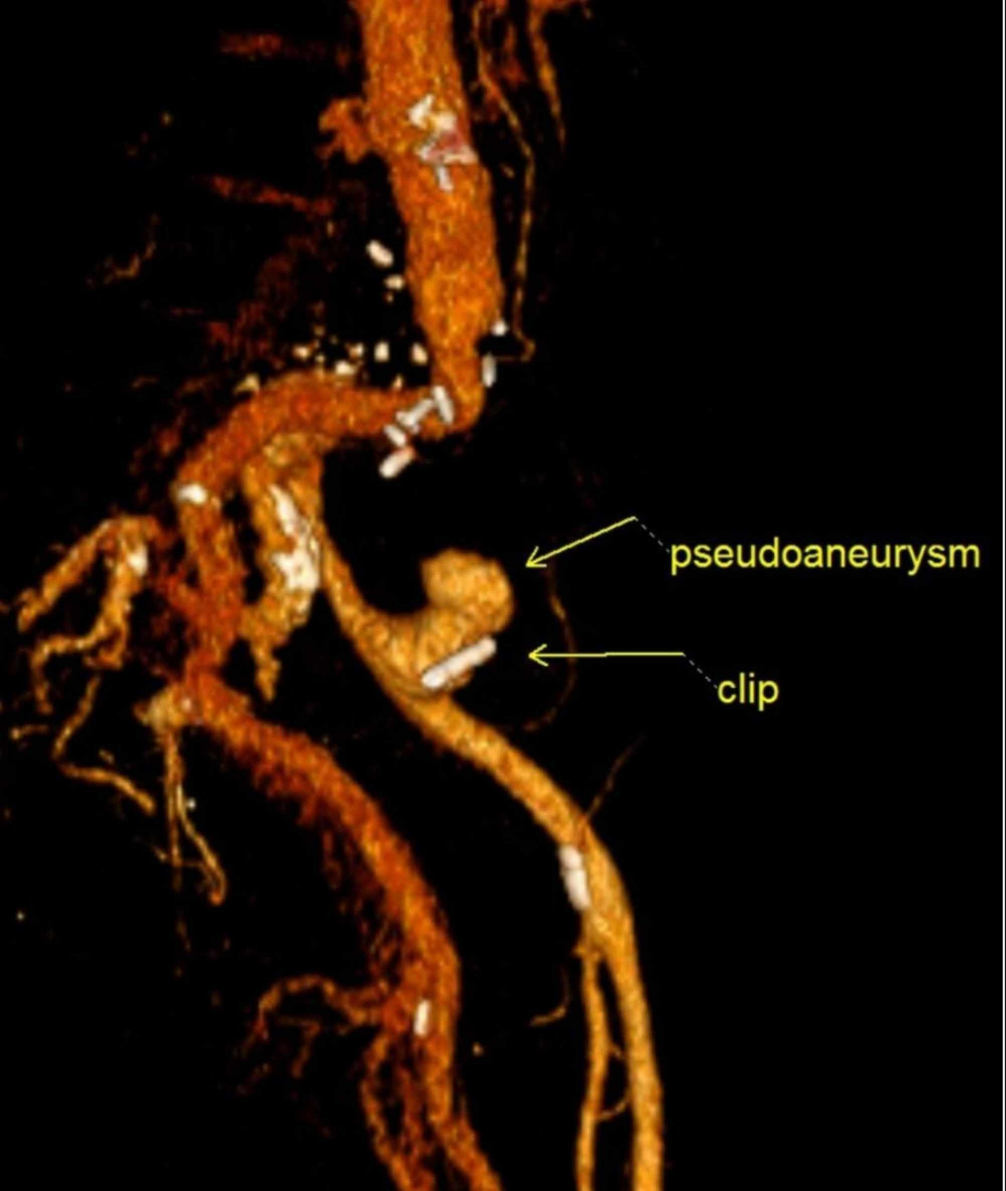
3D rendered computed tomography (CT) image demonstrates the endoscopically placed clip adjacent to the pseudoaneurysm; prior clips from previous aortoiliac surgery can also be seen

She was treated with large volume resuscitation, pantoprazole, and octreotide drips and underwent an emergent esophagogastroduodenoscopy (EGD) which was negative for pathology or bleeding. Colonoscopy revealed a 5 cm, pulsatile, submucosal mass in the sigmoid colon, with an actively bleeding ulcerated area (Figure 1). Epinephrine was injected with temporary hemostasis. A single hemoclip was placed to mark the site for potential embolization, as it was thought the lesion was not amenable for definitive endoscopic intervention. Differentials included ulcerated subepithelial mass like gastrointestinal stromal tumor or vascular erosion. A stat computed tomography (CT) angiogram of the abdomen and pelvis revealed a large left external iliac artery (LEIA) pseudoaneurysm in the region of the clips placed in the sigmoid colon during colonoscopy, occlusion of the proximal left common iliac artery (CIA), and left limb of aorto-bi-iliac (ABI) bypass (Figures 2-3). 

It appeared the patient had an open ABI bypass 15 years ago for a large distal AAA. She had a 16 x 18 mm bifurcated hemi shield graft end-to-end to the aorta and end-to-end to the right iliac artery and end-to-side to the left iliac artery. The etiology of SAEF was possibly related to previous clamp placement for ABI repair or distal anastomotic pseudoaneurysm. She underwent emergent endovascular repair of the LEIA pseudoaneurysm, with the placement of a LEIA stent graft. 

Intraoperative findings included patent left distal CIA with mild aneurysmal changes, patent left internal iliac artery, and patent LEIA with a large pseudoaneurysm projecting anteriorly. Occlusion of the patient's proximal left CIA and left limb of the ABI bypass allowed only retrograde access to the LEIA. Given the patient's significant herald bleed, tenuous clinical status, large body habitus, multiple medical comorbidities with poor functional status, and history of previous ABI bypass, the decision was made to approach the LEIA pseudoaneurysm endovascularly, despite the possible presence of vessel-enteric fistula. There was no evidence of bleeding into the GI tract or rupture of the pseudoaneurysm. There was difficulty in clearly visualizing the precise origin of the LEIA pseudoaneurysm. A 9 mm x 5 cm Viabahn stent graft (Gore Medical, Flagstaff, AZ) was inserted in the LEIA covering the area of the LEIA pseudoaneurysm. Post stent placement, balloon dilatation was performed using 10 mm x 4 cm balloon at both proximal and distal seal zones. Angiogram after balloon angioplasty did demonstrate some improvement, however, there was an area of possible leak. Due to limited oblique views in the operating room, limited working length available in the LEIA, as well as the fact that the patient was systemically heparinized for the procedure, we thought the small amount of continued flow into the pseudoaneurysm would likely resolve with reversal of anticoagulation and time, so the decision was made to not perform any additional stent placement.

**Figure 4:**
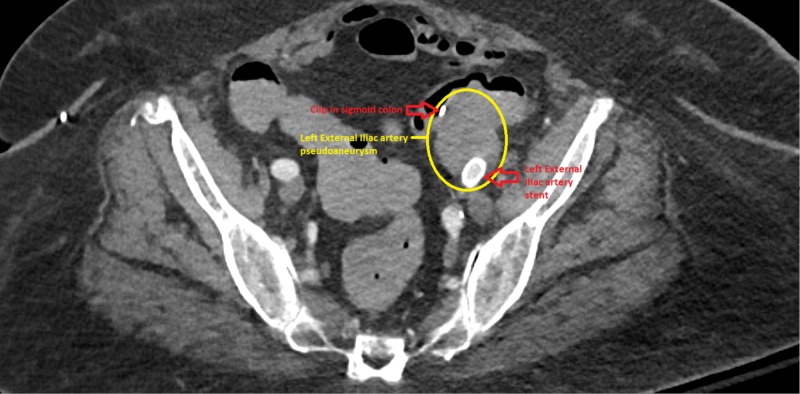
Post-operative computed tomography (CT) angiography of abdomen and pelvis shows patent left external iliac artery stent, with no endovascular leak

Repeat CT angiography of the abdomen demonstrated occlusion of the distal left CIA with reconstitution distally, likely from the internal iliac artery, placement of a patent LEIA stent and occlusion of the previously demonstrated saccular pseudoaneurysm extending off the anterior LEIA with no endoleak (Figure 4).

At the time, there was no obvious indication of infection of the patient's occluded left iliac bypass graft. However, it was understood that the patient likely had an underlying graft infection, given the fistulous connection between the pseudoaneurysm and colon. After discussion with the medicine and infectious disease teams, we decided not to initiate antibiotics. The patient did well post operatively and was discharged home. 

**Figure 5:**
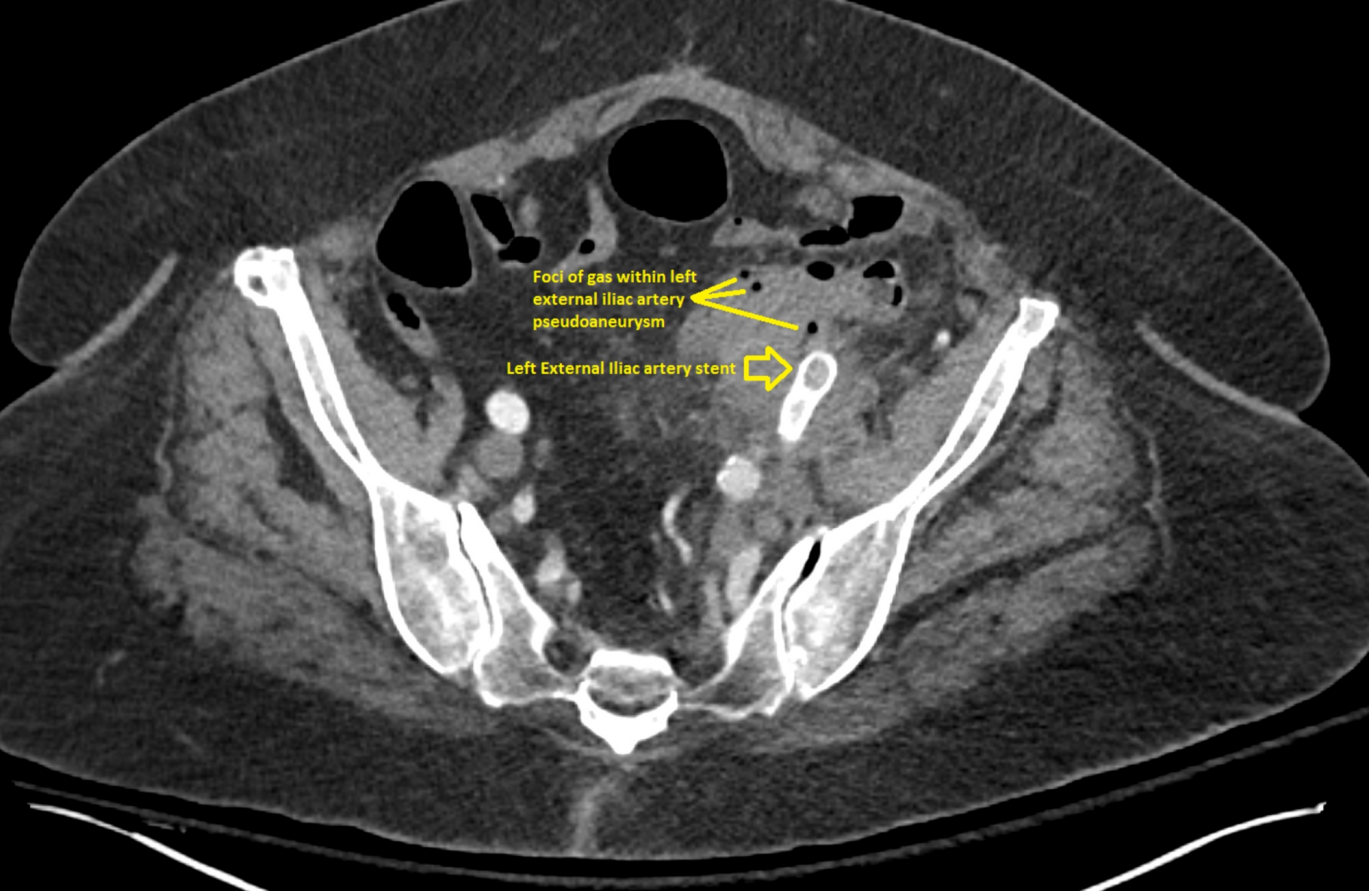
Computed tomography (CT) angiogram of the abdomen and pelvis showing patent left external iliac artery stent with foci of gas within the left external iliac artery pseudoaneurysm

She presented six months later with symptoms of nausea, vomiting, and vague abdominal pain that prompted a CT abdomen to be done. The CT findings reported new areas of gas/air consistent with infection at the site of the left limb of ABI graft. The LEIA stent-graft was patent, and there were some areas of mural thrombus in the stent-graft and possible trace extravasation into the pseudoaneurysm at the distal aspect of stent, however, the previous pseudoaneurysm with known connection to sigmoid colon, remains thrombosed, with no free retroperitoneal/intraperitoneal bleeding (Figure 5).

Based on these findings, treatment of the infected limb of the ABI graft would require complete ABI graft explant with aortic ligation and extra-anatomic bypass. Given the patient’s marginal functional status and the extent of the operation, there was a high risk of mortality. After discussion with the patient and her family, the patient declined major surgery and opted for symptomatic treatment with the possibility of extending the existing LEIA stent as a temporizing maneuver, depending on her clinical course. She was discharged home with hospice, followed up in a vascular clinic and declined palliative stent placement. 

She returned a month later with recurrent episodes of bright red blood per rectum and passage of clots. She underwent repeat CT angiography of the abdomen and pelvis, which demonstrated possible leak into the pseudoaneurysm from the distal aspect of the previously placed stent graft. With the understanding that this was essentially a palliative procedure, the patient eventually agreed to additional stent graft placements, as she continued to return to the hospital on multiple occasions with recurrent bleeding episodes. She underwent a palliative left iliac angiogram with stent placement. Intraoperatively retrograde left iliac angiogram demonstrated contrast extravasation into the pseudoaneurysm with some connection into the colon with contrast visualized in the bowel. The left internal iliac artery was patent. The previously placed LEIA stent and proximal LEIA were patent. She underwent successful stent placement essentially covering the entire LEIA with an 11 mm x 10 cm Viabahn stent graft placed proximally and 11 mm x 5 cm Viabahn stent graft placed distally. Completion angiogram demonstrated no further contrast extravasation into the bowel. She was discharged to a nursing home with plans to complete six weeks of intravenous (IV) piperacillin/tazobactam. 

She was last seen in the clinic one year after her initial presentation and was doing well with no recurrent bleeding or infections. 

## Discussion

AEF is an important differential to consider in older patients who present with large volume hematochezia. The most common symptom of AEF is GI bleeding (e.g., melena, a herald bleed, coffee ground emesis, bright red blood per rectum, severe hemorrhagic shock). Other rare symptoms of SAEF are fever and sepsis, pulsating abdominal mass, groin mass, retroperitoneal abscess, limb ischemia, abdominal pain, back pain, and weight loss [2]. The presence of prior signs of abdominal surgery should raise an index of suspicion for SAEF as it is difficult to diagnose considering its rarity. The rate of AEF occurrence in the colon is about 5% and there is limited data regarding survival [7,9]. Important risk factors to consider include aneurysms of the aorta or aortoiliac vessels and prior aortic intervention or surgery. Other intraabdominal processes that cause inflammation, infection or mechanical erosion of the aorta and adjacent structures can also lead to AEF. CT and EGD are frequently used to diagnose AEFs [10]. 

Without treatment AEF often proves to be fatal. Treatment of AEF includes volume resuscitation, antibiotics, and aortic repair. The surgical approach (open vs endovascular) for AEF repair should be individualized based on the clinical picture, type of AEF, timing of presentation, patient`s comorbidities and aortic anatomy. 

Given our patient's significant herald bleed with tenuous clinical status, large body habitus, medical comorbidities, poor functional status as well as previous ABI bypass, the decision was made to approach the pseudoaneurysm endovascularly, despite the possible presence of vessel-enteric fistula, as she was deemed unstable for an open approach. At her initial presentation, there was low suspicion of infection and antibiotics were not administered, subsequent presentation six months later demonstrated clinical infection but patient declined surgery and opted for home hospice care. Eventually, with recurrent episodes of GI bleed, she agreed to palliative stent placement at which time she was given a six-week course of IV antibiotics. She had a complicated course with multiple recurrent bleeds; each bleed increased her risk of mortality, however, she had successful outcomes. 

## Conclusions

In conclusion, the diagnosis of SAEF, a rare, life-threatening complication of AAA repair, depends on the clinical and radiographic findings. Early diagnosis is difficult and remains dependent on a heightened clinical suspicion. Surgical management depends on variable factors for optimal safe outcomes. There is high mortality associated with this entity and outcomes are usually not expected to be favorable with repeat presentations.
